# Supplementary material for: Muscle Strength Explains the Protective Effect of Physical Activity against COVID-19 Hospitalization among Adults aged 50 Years and Older
Source: medRxiv. 2021 Mar 1:2021.02.25.21252451. Preprint. [Version 1] doi: 10.1101/2021.02.25.21252451 (PMC7941660; doi:10.1101/2021.02.25.21252451)
Supplement: 1 [file NIHPP2021.02.25.21252451-supplement-1.pdf]

medRxiv preprint doi: https://doi.org/10.1101/2021.02.25.21252451; this version posted March 1, 2021. The copyright holder for this preprint (which was not certified by peer review) is the author/funder, who has granted medRxiv a license to display the preprint in perpetuity.
It is made available under a CC-BY 4.0 International license .

Supplementary material

Table S1. Results of the general logistic models testing the associations of low-to-moderate physical activity and other risk factors with COVID-19 hospitalizations.

Table S2. Results of the general logistic models testing the associations of vigorous physical activity and other risk factors with COVID-19 hospitalizations.

Table S3. Results based on the rare-events logistic regression with a tau parameter of 84/100,000.

Table S1. Results of the general logistic models testing the associations of low-to-moderate physical activity and other risk factors with COVID-19 hospitalizations.

	Model 0			Model 1			Model 2		
**Predictors**	**OR**	**95CI**	***p***	**OR**	**95CI**	***p***	**OR**	**95CI**	***p***
Intercept	0.05	0.03 – 0.09	<.001	0.02	0.01 – 0.05	<.001	0.01	0.00 – 0.03	<.001
Low-to-moderate Physical activity (Ref: *Hardly ever or never*)									
Once to three times a month	0.66	0.21 – 1.82	.442	0.68	0.21 – 1.89	.477	0.85	0.26 – 2.44	.778
Once a week	0.59	0.28 – 1.16	.146	0.64	0.30 – 1.27	.226	0.78	0.36 – 1.56	.503
More than once a week	0.31	0.11 – 0.78	.016	0.33	0.12 – 0.84	.023	0.42	0.15 – 1.09	.081
Age (Ref: 50-64 years)									
65-74 years				2.25	1.06 – 5.22	.043	1.91	0.88 – 4.48	.112
75-96 years				4.64	2.29 – 10.40	<.001	3.28	1.55 – 7.61	.003
Sex (Ref: *Women*)				0.75	0.38 – 1.45	.395	1.17	0.54 – 2.52	.688
Height				1.22	0.86 – 1.73	.252	1.45	1.01 – 2.08	.042
Muscle strength							0.59	0.40 – 0.89	.011
Body Mass Index (Ref: *Normal weight*)									
Obese							1.81	0.90 – 3.59	.092
Overweight							1.46	0.81 – 2.68	.212
Lung Disease (Ref: *No*)							1.53	0.61 – 3.28	.314
Cardiovascular disease (Ref: *No)*							1.07	0.62 – 1.88	.800
Diabetes (Ref: *No*)							1.08	0.50 – 2.14	.830
Cancer (Ref: *No*)							0.80	0.19 – 2.26	.712
Arthritis (Ref: *No*)							1.27	0.58 – 2.51	.524
Kidney Disease (Ref: *No*)							2.18	0.49 – 6.69	.227

Note: Odds ratios (OR), 95% Confidence Intervals (95CI), and *p*-values (*p*) are reported.

medRxiv preprint doi: https://doi.org/10.1101/2021.02.25.21252451; this version posted March 1, 2021. The copyright holder for this preprint **(which was not certified by peer review)** is the author/funder, who has granted medRxiv a license to display the preprint in perpetuity.
It is made available under a CC-BY 4.0 International license .

Table S2. Results of the general logistic models testing the associations of vigorous physical activity and other risk factors with COVID-19 hospitalizations.

	Model 0			Model 1			Model 2		
**Predictors**	**OR**	**95CI**	***p***	**OR**	**95CI**	***p***	**OR**	**95CI**	***p***
Intercept	0.03	0.02 – 0.04	<.001	0.01	0.01 – 0.03	<.001	0.01	0.00 – 0.02	<.001
Vigorous Physical activity (Ref: *Hardly ever or never*)									
Once to three times a month	0.66	0.22 – 1.55	.387	0.71	0.24 – 1.70	.491	0.85	0.29 – 2.07	.751
Once a week	0.65	0.30 – 1.28	.240	0.71	0.33 – 1.41	.354	0.87	0.40 – 1.75	.711
More than once a week	0.40	0.21 – 0.71	.003	0.46	0.24 – 0.83	.012	0.61	0.31 – 1.13	.121
Age (Ref: *50-64 years*)									
65-74 years				2.20	1.03 – 5.09	.051	1.86	0.86 – 4.36	.127
75-96 years				4.44	2.19 – 9.98	<.001	3.18	1.51 – 7.38	.004
Sex (Ref: *Women*)				0.77	0.39 – 1.48	.428	1.18	0.54 – 2.54	.671
Height				1.23	0.87 – 1.74	.233	1.46	1.01 – 2.08	.041
Muscle strength							0.59	0.39 – 0.89	.011
Body Mass Index (Ref: *Normal weight*)									
Obese							1.80	0.90 – 3.58	.093
Overweight							1.45	0.81 – 2.66	.220
Lung Disease (Ref: *No*)							1.50	0.60 – 3.25	.338
Cardiovascular disease (Ref: *No*)							1.08	0.63 – 1.88	.777
Diabetes (Ref: *No*)							1.09	0.50 – 2.14	.822
Cancer (Ref: *No*)							0.78	0.18 – 2.20	.680
Arthritis (Ref: *No*)							1.26	0.58 – 2.51	.530
Kidney Disease (Ref: *No*)							2.07	0.47 – 6.42	.261

Note: Odds ratios (OR), 95% Confidence Intervals (95CI), and *p*-values (*p*) are reported.

medRxiv preprint doi: https://doi.org/10.1101/2021.02.25.21252451; this version posted March 1, 2021. The copyright holder for this preprint (which was not certified by peer review) is the author/funder, who has granted medRxiv a license to display the preprint in perpetuity.
It is made available under a CC-BY 4.0 International license .

Table S3. Results based on the rare-events logistic regression with a tau parameter of 84/100,000.

	Model 0			Model 1			Model 2		
**Predictors**	**OR**	**95CI**	***p***	**OR**	**95CI**	***p***	**OR**	**95CI**	***p***
Intercept	1.3E-2	9.1E-4 – 1.7E-3	<.001	5.7E-4	2.7E-4 – 1.2E-3	<.001	3.2E-4	1.3E-4 – 7.7E-4	<.001
Physical activity (Ref: *Hardly ever or never*)									
Once to three times a month	0.65	0.25 – 1.66	.364	0.69	0.27 – 1.80	.451	0.82	0.31 – 2.16	.695
Once a week	0.61	0.30 – 1.25	.176	0.67	0.33 – 1.36	.263	0.80	0.38 – 1.64	.550
More than once a week	0.42	0.23 – 0.77	.005	0.49	0.27 – 0.89	.019	0.65	0.35 – 1.22	.179
Age (Ref: *50-64 years*)									
65-74 years				2.13	0.97 – 4.69	.060	1.81	0.81 – 4.03	.147
75-96 years				4.25	2.03 – 8.87	<.001	2.99	1.36 – 6.56	.006
Sex (Ref: *Women*)				0.77	0.37 – 1.60	.480	1.22	0.54 – 2.75	.633
Height				1.23	0.88 – 1.73	.231	1.47	1.01 – 2.14	.047
Muscle strength							0.57	0.37 – 0.89	.013
Body Mass Index (Ref: *Normal weight*)									
Obese							1.80	0.89 – 3.61	.101
Overweight							1.47	0.82 – 2.66	.199
Lung Disease (Ref: *No*)							1.57	0.68 – 3.60	.290
Cardiovascular disease (Ref: *No*)							1.07	0.63 – 1.83	.802
Diabetes (Ref: *No*)							1.14	0.54 – 2.38	.735
Cancer (Ref: *No*)							0.86	0.24 –3.14	.822
Arthritis (Ref: *No*)							1.32	0.66 – 2.63	.437
Kidney Disease (Ref: *No*)							2.55	0.73 – 8.83	.141

Note: Odds ratios (OR), 95% Confidence Intervals (95CI), and *p*-values (*p*) are reported.

medRxiv preprint doi: https://doi.org/10.1101/2021.02.25.21252451; this version posted March 1, 2021. The copyright holder for this preprint (which was not certified by peer review) is the author/funder, who has granted medRxiv a license to display the preprint in perpetuity.
It is made available under a CC-BY 4.0 International license .
